# Cubic three-dimensional hybrid silica solids for nuclear hyperpolarization†
†Electronic supplementary information (ESI) available. See DOI: 10.1039/c6sc02055k



**DOI:** 10.1039/c6sc02055k

**Published:** 2016-07-18

**Authors:** D. Baudouin, H. A. van Kalkeren, A. Bornet, B. Vuichoud, L. Veyre, M. Cavaillès, M. Schwarzwälder, W.-C. Liao, D. Gajan, G. Bodenhausen, L. Emsley, A. Lesage, S. Jannin, C. Copéret, C. Thieuleux

**Affiliations:** a Université de Lyon , Institut de Chimie de Lyon , LC2P2 , UMR 5265 CNRS-CPE Lyon-UCBL , CPE Lyon , 43 Bvd du 11 Novembre 1918 , 69100 Villeurbanne , France . Email: david.baudouin@univ-lyon1.fr ; Email: chloe.thieuleux@univ-lyon1.fr; b Institut des Sciences et Ingénierie Chimiques , Ecole Polytechnique Fédérale de Lausanne (EPFL) , CH-1015 Lausanne , Switzerland; c ETH Zürich , Department of Chemistry and Applied Biosciences , Vladimir-Prelog-Weg 1-5/10 , 8093 Zürich , Switzerland . Email: ccoperet@inorg.chem.ethz.ch; d Université de Lyon , Institut des Sciences Analytiques , UMR 5280 , CNRS , Université Lyon 1 , ENS Lyon 5 rue de la Doua , F-69100 Villeurbanne , France; e Département de Chimie , Ecole Normale Supérieure, 24 Rue Lhomond , 75231 Paris Cedex 05 , France; f Université Pierre-et-Marie Curie , Paris , France; g UMR 7203 , CNRS/UPMC/ENS , Paris , France

## Abstract

Hyperpolarization of metabolites by dissolution dynamic nuclear polarization (D-DNP) for MRI applications often requires fast and efficient removal of the radicals (polarizing agents). Ordered mesoporous SBA-15 silica materials containing homogeneously dispersed radicals, referred to as HYperPolarizing SOlids (HYPSOs), enable high polarization – *P*(^1^H) = 50% at 1.2 K – and straightforward separation of the polarizing HYPSO material from the hyperpolarized solution by filtration. However, the one-dimensional tubular pores of SBA-15 type materials are not ideal for nuclear spin diffusion, which may limit efficient polarization. Here, we develop a generation of hyperpolarizing solids based on a SBA-16 structure with a network of pores interconnected in three dimensions, which allows a significant increase of polarization, *i.e. P*(^1^H) = 63% at 1.2 K. This result illustrates how one can improve materials by combining a control of the incorporation of radicals with a better design of the porous network structures.

## Abstract 2

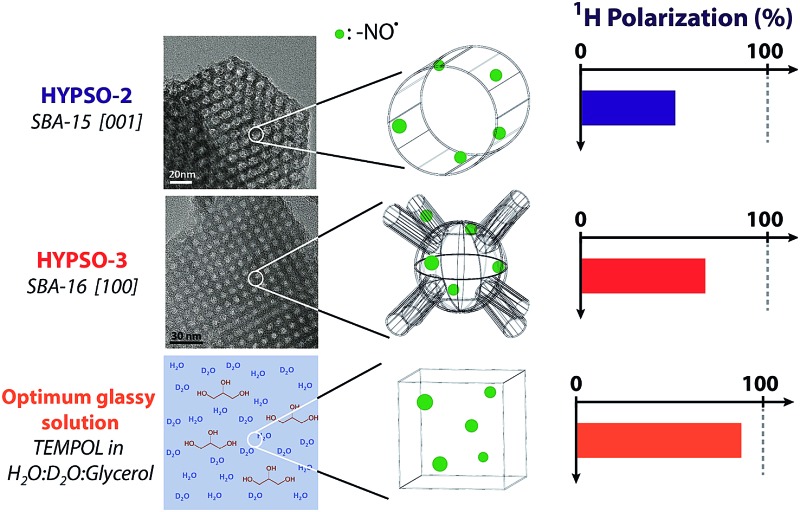
Porous network architecture of hybrid silicas containing TEMPO radicals along their pores is key for increased hyperpolarization performances.

One of the major limitations of nuclear magnetic resonance spectroscopy (NMR) and magnetic resonance imaging (MRI) is their intrinsic low sensitivity, which arises from low thermal equilibrium (Boltzmann) polarization of the nuclear spins at room temperature, even with the highest field instruments. This low sensitivity is particularly challenging for low-gamma nuclear spins. For carbon-13, polarization is as low as *P*(^13^C) ≈ 2 × 10^–5^ at *T* = 300 K and *B*_0_ = 23.5 T. This weakness can be circumvented by dissolution dynamic nuclear polarization (D-DNP).^1,2^ D-DNP takes advantage of the high electron spin polarization at low temperatures to enhance the nuclear spin polarization well beyond thermodynamic equilibrium (>10 000 times) *via* microwave irradiation. Such huge gains in sensitivity allow metabolic imaging and have for example enabled the detection of anomalous metabolic rates in prostate tumors in living patients.^3^

Hyperpolarization by D-DNP involves microwave irradiation at low temperatures in moderate magnetic fields (typically *T* = 1.2 K and *B*_0_ = 3.35 or 6.7 T) of frozen glassy solutions doped with stable free radicals and molecules of interest (*e.g.* metabolites or tracers). The preparation of a glassy frozen sample is important because it ensures that the radicals are statistically distributed, without the formation of ice crystals, which leads to optimal DNP efficiency. For that purpose, glass-forming agents such as glycerol, DMSO or ethanol are usually included in high concentrations. In a typical D-DNP experiment, following polarization, the polarized solution is rapidly brought to room temperature using superheated water and quickly transferred to the MRI or NMR machine for further studies. Once the solution is hyperpolarized, both radicals and glass-forming agents are unwanted and should obviously not be injected into patients. Furthermore, radicals act as paramagnetic relaxing agents, inducing faster depolarization.^4^ Therefore, rapid removal of radicals after polarization and before use is essential. In the case of trityl and BDPA radicals, the removal can be achieved by precipitation followed by filtration or by ion exchange.^5,6^ However both methods are limited to specific sample formulations. Alternatively, nitroxide based radicals can be scavenged by ascorbate (vitamin C),^7^ which attenuates paramagnetic relaxation but leads to contamination of the samples by hydroxylamines. Radicals can be incorporated into polymers such as polystyrene particles^8^ or hydrogels,^9^ allowing physical separation of the polarizing agent from the solution, but the efficiency for D-DNP system is limited and the filtration not straightforward.

In this context, we have recently developed a family of solid polarizing matrices based on hybrid materials containing covalently bound radicals, coined HYperPolarizing SOlids (HYPSO). These materials provide in principle a universal solution to the above-mentioned issues: *i.e.* fast and easy removal of radicals by filtration, and the absence of glass-forming agents. HYPSO are porous and robust silica-based solids on which any radicals (*i.e.* TEMPO, trityl…) can be covalently and homogeneously attached to the surface of their pores.^10,11^ We showed an efficient direct polarization approaching *P*(^13^C) = 15% with a build-up time of 2 h in a 3 M 1-^13^C sodium pyruvate aqueous solution impregnated with trityl-based HYPSO.^11^ The first generation TEMPO-based materials, HYPSO-1, allowed reaching a ^13^C polarization as high as *P*(^13^C) = 33% in only 20 min using a state-of-the-art polarizer including microwave frequency modulation^12^ and ^1^H-^13^C cross-polarization.^13^ Despite significant research efforts to improve the DNP performances of the first generations of HYPSOs, it was not possible to enhance the proton polarization beyond *P*(^1^H) = 50%, well below the *P*(^1^H) = 90% that can be obtained under similar conditions in glassy water/glycerol TEMPOL solutions.^13^

The two first generations of HYPSOs were based on ordered mesoporous SBA-15 type structures, with a skeleton consisting of 8–10 nm diameter 1D-pore channels stacked in a 2D-hexagonal arrangement (Fig. 1 – top right). Two generations of nitroxide-based materials, HYPSO-1 and HYPSO-2, were hence prepared and differ from the linker used to anchor the radical to the solid surface, a propylamido^10^ and a 1,2,3-triazole-propyl tethers, respectively.^11^ Using a direct synthesis, the radicals were homogeneously incorporated onto the pore surface of HYPSO-1 and HYPSO-2 by peptide coupling or click chemistry, respectively, avoiding radical aggregation, which is important for D-DNP. However, in such a structure, the pores do not communicate with each other; we thus hypothesized that this could be a limiting factor for both nuclear spin diffusion and for the three-dimensional distribution of the radicals, in comparison to frozen glassy solutions. We therefore reasoned that a silica architecture with a 3D cubic porous network (for example using SBA-16 like structures) could improve the DNP performance. Here we describe the development of materials with cubic network arrangement (HYPSO-3) and show that they lead to greater polarization with respect to the one-dimensional porous HYPSO-1/2 materials, yielding proton polarization up to *P*(^1^H) = 63%. We show how the 3D cubic material can also be efficiently used under Magic Angle Spinning (MAS) DNP conditions.

**Fig. 1:**
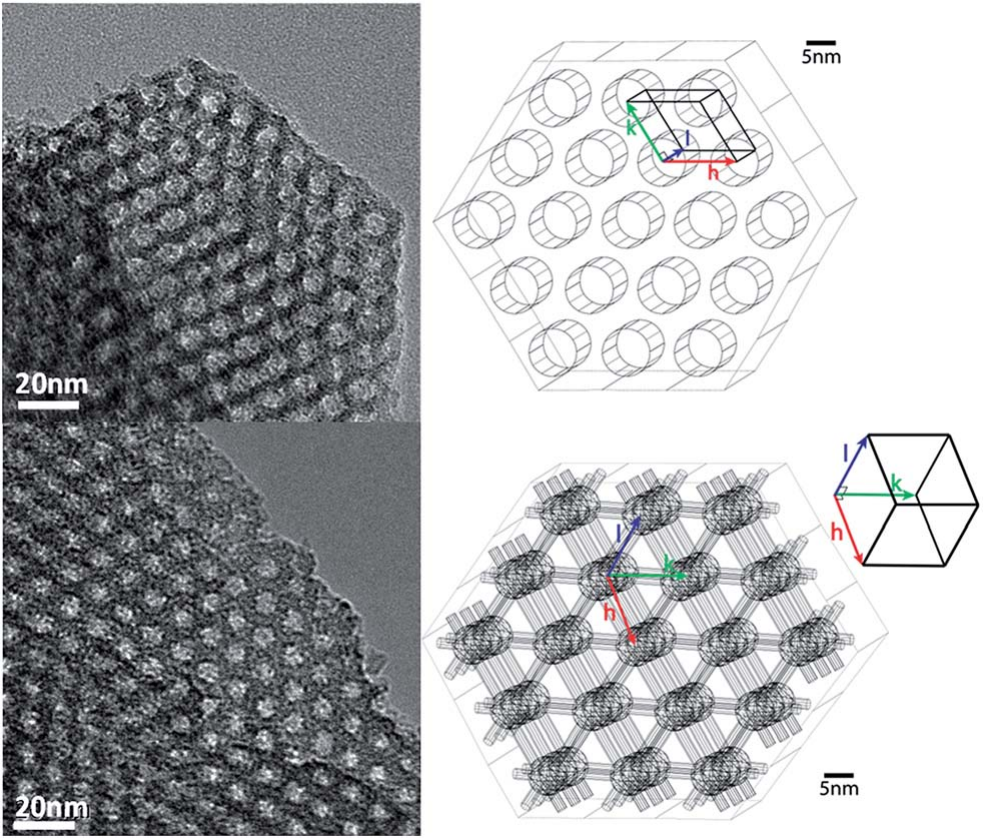
Left: TEM pictures of hexagonal 1/100_N_3__SBA-15 in the [001] axis (top) and of cubic 1/140_N_3__SBA-16 in the [111] axis (bottom). Right: Schematic representations of the 2D pore structure in the [001] axis of SBA-15/HYPSO-2 (top) and the 3D pore structure in the [111] axis of SBA-16/HYPSO-3 (axis). The radicals are distributed uniformly over the surface of the pores. See Fig. SI-1† for SBA-16 in [100] axis.

## Results and discussion

### Preparation and characterization of materials

First, propylazido functionalized SBA-16 materials were prepared by co-condensation of (3-azidopropyl)triethoxylsilane and tetraethyl orthosilicate in an HCl/NaCl aqueous medium in the presence of pluronic F127 as a structure-directing agent (SDA).^14^ The ratios between (3-azidopropyl)triethoxylsilane and tetraethyl orthosilicate varied in the range 1/34, 1/60, 1/100, 1/140, 1/320, corresponding to a loading of organic functionalities of 472, 272, 164, 118 and 52 μmol_≡SiR_ g^–1^. The materials obtained were filtered, and the SDA was extracted with a pyridine/HCl solution for 24 h and finally washed and dried under 10^–5^ mbar vacuum at 135 °C.^15^ The different materials were analyzed by N_2_ adsorption, which showed a type IV isotherm characteristic of mesoporous materials. The BET model indicated surface areas ranging from *ca.* 910 to 1080 m^2^ g^–1^ (see Table 1) with total pore volumes (*P*/*P*_0_ < 0.99) of 0.62–0.82 cm^3^ g^–1^, of which 37–46% are micropores according to the α-plot model. The pore sizes have narrow bimodal distributions at 1.4–1.7 (inter-connecting windows, MP model) and 6.2–7.0 nm (cavities, BJH model). The sharp drop in the desorption branch of the isotherm observed at *ca.* 0.45 *P*/*P*_0_ is typical of nitrogen desorption from mesoporous materials through smaller pores (ink bottle effect), in line with the formation of 6–7 nm cages interconnected by 1–2 nm connecting micropores. Small Angle X-Ray Diffraction (SA-XRD, see Table SI-1†) of 1/34_N_3__SBA-16 presented a strong peak at *ca.* 2*θ* = 0.75° assigned to the X-ray diffraction of the {110} family of planes, indicative of well-structured cubic-centered body SBA-16 materials (*Im*3*m* space group). SA-XRD and N_2_ adsorption/desorption data allow one to calculate a mean micropore length of *ca.* 10 nm (Table 1 and SI-1†). Transmission Electron Micrographs (TEM) of 1/140_N_3__SBA-16 confirm the formation of a cubic ordered mesoporous material (see representative pictures in Fig. 1 and S1†).

**Table 1:** Organic function loadings and textural characteristics of the materials obtained from SA-XRD and N_2_-adsorption desorption at 77 K

Materials	[≡SiR]/μmol_≡SiR_ g^–1^	*S*_BET_/m^2^ g^–1^	*V*_p_ ^*a*^ (tot.)/m^2^ g^–1^	*V*_p_ ^*b*^ (μ)/m^2^ g^–1^	*D*_p_ ^*c*^/nm	*L*_μpore_ ^*d*^ /nm
1/34_N_3__SBA-16	472	1012	0.68	0.31	1.7/6.2	9.6
1/34_HYPSO-3	472	729	0.50	0.19	1.6/5.4	10.4
1/60_N_3__SBA-16	272	1010	0.66	0.29	1.6/6.3	n.d.
1/60_HYPSO-3	272	752	0.52	0.22	1.6/5.4	10.2
1/100_N_3__SBA-16	164	913	0.62	0.11	1.7/6.2	n.d.
1/100_HYPSO-3	164	893	0.63	0.26	1.3/6.3	8.6
1/140_N_3__SBA-16	118	1184	0.82	0.33	1.4/7.0	n.d.
1/140_HYPSO-3	118	983	0.69	0.26	1.7/7.1	9.3
1/320_N_3__SBA-16	52	1068	0.75	0.48	1.7/7.0	n.d.
1/320_HYPSO-3	52	714	0.48	0.27	1.6/5.4	12.3

^*a*^Total pore volume corresponding to the quantity of N_2_ adsorbed at *P*/*P*_0_ = 0.99.

^*b*^Micropore volume, calculated from the α_S_ plot model.

^*c*^Micropore mean diameter calculated using MP model/mesopore mean diameter calculated using the BJH model (adsorption branch).

^*d*^Micropore mean length, calculated using *L*_μpore_ = (*d*_(110)_/cos(π/4) – *D*_meso_) using the mesoporous diameter *D*_p_ and the *d*-spacing *d*(110) obtained from Small Angle XRD analysis.

For comparison, several polarizing matrices with 2D hexagonal arrangements of mesopore tube-like pores (SBA-15 type materials), hereafter named HYPSO-2, were also prepared.^10^ These materials are highly porous with a BET surface area, a total pore volume, BJH and MP pore diameters of 770–870 m^2^ g^–1^, 1.1–1.2 cm^3^ g^–1^ and 8.0–9.2 nm, respectively (Table S2†).

Importantly, these SBA-15 type materials exhibit non-interconnected 1.8 nm micropores (5–8% of pore volume). In contrast, HYPSO-3 (SBA-16 materials) exhibits spherical mesopores interconnected by micro-channels in all three dimensions. This pore structure leads to different textures: a lower pore volume and a different intra-grain pore volume distribution (Fig. 1).

Post-functionalization of N_3__SBA-16 to obtain HYPSO-3 materials was performed using copper-catalyzed azide-alkyne cycloaddition (Cu-AAC)^16^ in the presence of *o*-propargyl TEMPO, CuI, dry DMF and Et_3_N (see details in ESI and Fig. S2 and S3†). Diffuse Reflectance Infrared Fourier Transform Spectroscopy (DRIFTS) analysis of the powder allowed evaluation of the efficiency of the cycloaddition (referred to as Cu-AAC yield) on HYPSO-3.

As shown in Table 2, 88–64% of the starting azido –N_3_ reacted with the *o*-propargyl TEMPO reactant, the yield decreasing slowly when decreasing the molar concentration of radicals (quoted 1/*xx* ratio that stands for 1 mol of radical per *xx* mol of SiO_2_). The concentration of radical incorporated in HYPSO-3 was quantified by recording X-band CW EPR spectra at room temperature. Nitroxyl radical loadings of 246, 135, 79, 50 and 33 μmol_NO_ g^–1^ were measured for ratios of 1/34, 1/60, 1/100, 1/140 and 1/320 respectively, corresponding to radical concentrations of 491, 260, 125, 72 and 67 μmol_NO_ cm^–3^ within the total volume of the pores. The radical concentrations show that the yields of post-functionalization are in the range 41–63%, similar to 2D-hexagonal materials characterized by the same method (42–57%). No significant difference between the EPR profiles of HYPSO-2 and HYPSO-3 could be observed at room temperature (Fig. S4†).

**Table 2:** Characteristics of HYPSO-3 materials

Ratio	[R]/μmol g^–1^	[NO˙]/μmol g^–1^	Cu-_AAC_ yield^*a*^ (%)	EPR yield^*b*^ (%)	[NO˙]^*c*^ /μmol cm^–3^
1/34	472	246	88	52	491
1/60	272	135	81	50	260
1/100	164	79	77	48	125
1/140	118	50	74	41	72
1/320	52	33	64	63	67

^*a*^Percentage of N_3_ reacted after Cu-AAC (obtained by DRIFT).

^*b*^Percentage of NO˙ compared to initial N_3_.

^*c*^Concentration per total pore volume (*P*/*P*_0_ = 0.99).

### EPR spectroscopy

**Fig. 2:**
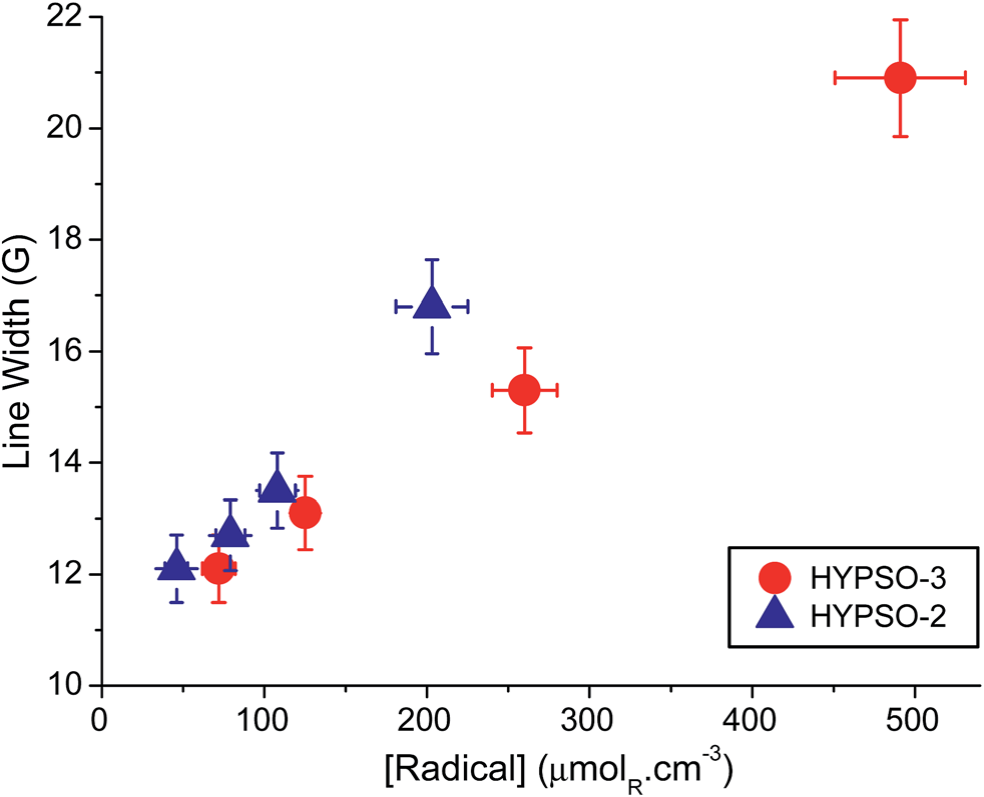
EPR linewidths of HYPSO-2 and -3 as a function of the molar radical concentration (in μmol_NO_ cm^–3^).

The average inter-radical distances in HYPSO-3 were evaluated from the line width of the central EPR signal at 110 K, which is known to be broadened by electron–electron dipolar couplings and by spin exchange.^17^ Such an analysis is limited to an average inter-radical distance *r*_RR_ < 2 nm. For larger distances, the dipolar line width is masked by inhomogeneous broadening (≈12 G). The EPR line-widths measured are 20.9, 15.3, 13.1 and 12.1 G for HYPSO-3 with ratios N_3_/TEOS = 1/34, 1/60, 1/100 and 1/140 respectively (Fig. 2 and Table S3†). The dipolar broadening is almost proportional to the radical concentrations for HYPSO-2 and -3, although the slopes are different. Indeed, the line width of HYPSO-3 (SBA-16) is narrower than that of HYPSO-2 (SBA-15, data from ref. 11) for the same volumetric radical concentration. This narrower line width can be attributed to a more uniform 3D distribution of the radicals. We believe that this improvement likely originates from the difference in pore shape (tubular *vs.* interconnected cages) and structuration between HYPSO-2 and HYPSO-3.

### DNP performances

**Fig. 3:**
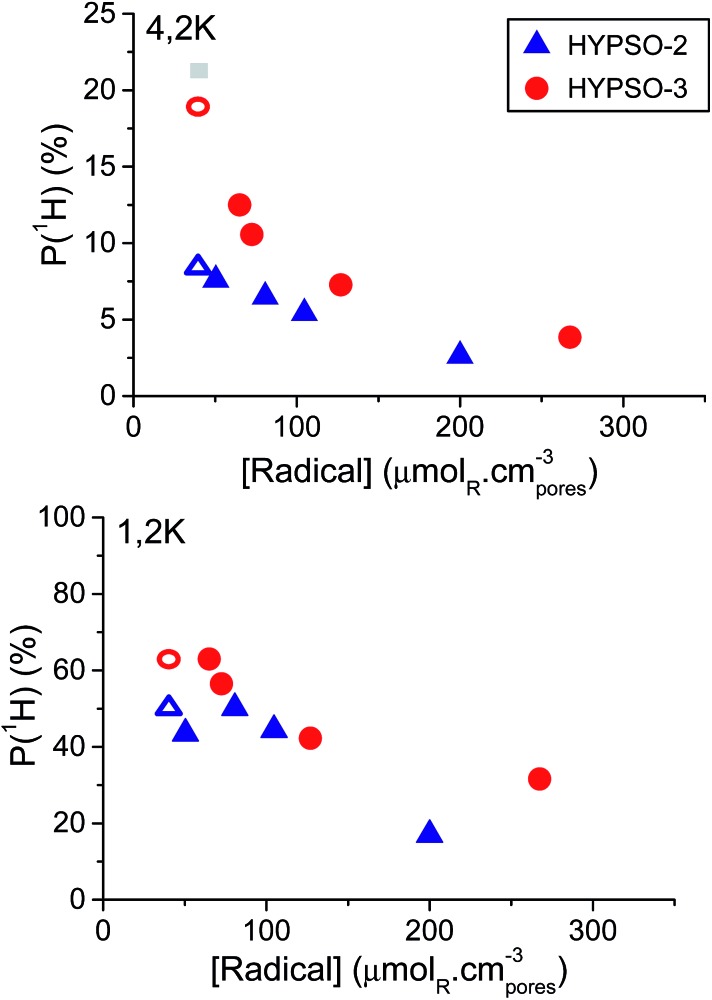
Polarization *P*(^1^H) measured using microwave frequency modulation at 4.2 K (top) and 1.2 K (bottom) and 6.7 T as a function of radical concentration (in μmol_NO_ cm^–3^) for: HYPSO-2 (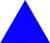) and HYPSO-3 (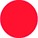) impregnated with D_2_O : H_2_O (8 : 2). The open circles 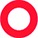 and triangles 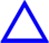 correspond to a solution of 40 mM TEMPOL in H_2_O : D_2_O : glycerol-d_8_ (10 : 40 : 50) impregnated in 1/140_N_3__SBA-16 (radical-free HYPSO-3) and 1/140_N_3__SBA-15 (radical-free HYPSO-2), respectively. The grey square 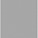 corresponds to a TEMPOL reference DNP solution, without HYPSO.

The DNP efficiency of HYPSO-3 was first evaluated for samples spinning at the magic angle (MAS) near 100 K and gave results similar to HYPSO-2 ^10,18,19^ (see ESI†). The DNP performance of HYPSO-2 and -3 was then determined at 4.2 and 1.2 K. Both materials were impregnated by filling *ca.* 95% of the pore volume with D_2_O : H_2_O (8 : 2). The results obtained at 4.2 K with microwave frequency modulation^12^ are presented in Fig. 3.

One can observe a maximum polarization in the vicinity of [R] = 50 μmol cm^–3^. At this concentration, HYPSO-3 yields a polarization *P*(^1^H) = 12.5%, significantly higher than the 7.5% obtained with HYPSO-2. The use of microwave frequency modulation improves the DNP performances of both HYPSO-2 and -3 but only for [R] < 100 μmol cm^–3^ (see Fig. S6†).

For comparison, an isotropic “glassy” H_2_O : D_2_O : glycerol-d_8_ (10 : 40 : 50) matrix without HYPSO doped with 40 mM TEMPOL (40 μmol_NO_ cm^–3^) gave rise to *P*(^1^H) = 21.5%. Note that a 80 μmol_NO_ cm^–3^ solution gives comparable results. When impregnating HYPSO-3 type matrices containing surface azido-groups instead of TEMPO units (1/140_N3_SBA-16) with a 40 mM TEMPO solution, a polarization *P*(^1^H) = 19% was obtained. This polarization value is close to that of the isotropic glassy DNP solution (*P*(^1^H) = 21.5%). On the contrary, when impregnated in a HYPSO-2 matrix without any radicals (1/140_N_3__SBA-15), the ^1^H polarization dropped to *P*(^1^H) = 9%. We take this as a strong indication that the cubic 3D porous network is advantageous for efficient DNP as compared to a one-dimensional network.

The build-up time constant (Fig. S6†) was found to be *τ*_DNP_ = 74 s for the reference DNP solution without HYPSO at 4.2 K. The same solution impregnated in 1/140_N_3__SBA-16 (same as HYPSO-3) gave *τ*_DNP_ = 145 s for a similar polarization, and *τ*_DNP_ = 77 s when impregnated in 1/140_N_3__SBA-15 (same as HYPSO 2) (Fig. S6†). EPR studies confirmed that this lengthening of the DNP build-up in HYPSO-3 was not due to a radical quenching effect.

At 1.2 K, HYPSO-3 yielded a polarization *P*(^1^H) > 40% over a broad range of radical concentrations 50 < [R] < 160 μmol_NO_ cm^–3^, reaching *P*(^1^H) = 63% for [R] = 67 μmol_NO_ cm^–3^ (*cf.* Fig. 3, bottom).

**Fig. 4:**
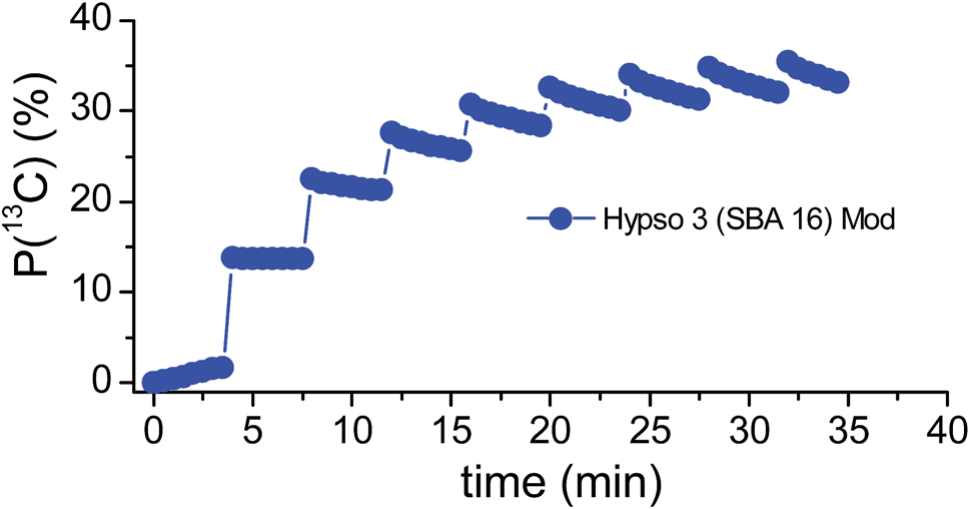
Left ^1^H → ^13^C CP-DNP performed on HYPSO-3 material (67 μmol_NO_ cm^–3^) impregnated with a 3 M solution of [1-^13^C]-acetate in D_2_O. *P*(^13^C) 36% is reached in 32.5 min with ^1^H → ^13^C CP applied at 4 min intervals.

By comparison, HYPSO-2 only yielded a maximum polarization *P*(^1^H) = 50% for an optimal [R] = 79 μmol_NO_ cm^–3^. The polarization in the H_2_O : D_2_O : glycerol-d_8_ (10 : 40 : 50) mixture containing 40 mM TEMPOL can reach *P*(^1^H) = 90% under the same conditions.^13^ At 1.2 K, frequency modulation was found to have a positive effect on DNP for HYPSO-2 and -3 when [R] < 75 μmol_NO_ cm^–3^. When the optimal DNP solution was impregnated in 1/140_N_3__SBA-16 (radical-free HYPSO-3) and 1/140_N_3__SBA-15 (radical-free HYPSO-2), we observed *P*(^1^H) = 63% for HYPSO-3 and 50% for HYPSO-2 at 1.2 K. Note that higher polarization might be reached using HYPSO-3 with lower radical concentration, but build up times (*τ*_DNP_) would become very long (>300 s) (see Fig. S6†). Finally, we prepared a 3 M solution of sodium [1-^13^C]-acetate in H_2_O : D_2_O (1 : 9) to impregnate HYPSO-3 (with [R] = 67 μmol_NO_ cm^–3^) and we obtained *P*(^1^H) ≈ 50%. Cross polarization^20^ was performed with 8 contacts every 4 min, yielding *P*(^1^H → ^13^C) = 36% after *ca.* 30 minutes (Fig. 4). After dissolution, the aqueous acetate solution was recovered by filtration and centrifugation and subjected to ESR analysis which confirmed the presence of a negligible quantity of radicals in the liquid (*ca.* 1 μmol L^–1^ *i.e.* <0.3% of HYPSO) probably arising from the presence of very small grains of HYPSO-3 in the solution.

## Conclusions

In conclusion, we have shown that the architecture of the porous network affects the hyperpolarization properties of HYPSO materials for DNP. Here, a material with 1D tubular pores (HYPSO-2) allows one to reach *P*(^1^H) = 50% at 1.2 K, while a material with 3D interconnected cage-like pores in cubic symmetry (HYPSO-3) leads to a polarization of *P*(^1^H) = 63%. We propose that this is due to (i) a more homogeneous 3D distribution of nitroxyl radicals in HYPSO-3, as demonstrated by EPR, and (ii) the interconnection of the pores, which allows for nuclear spin diffusion in all three directions, as in ideal solutions. We are currently working on designing optimal materials based on these underlying principles.
