# Supplementary material for: Cubic three-dimensional hybrid silica solids for nuclear hyperpolarization
Source: Chem Sci. 2016 Jul 18;7(11):6846–50. doi: 10.1039/c6sc02055k (PMC5356032; doi:10.1039/c6sc02055k)
Supplement: Supplementary file 1 [file SC-007-C6SC02055K-s001.pdf]

Electronic Supplementary Material (ESI) for Chemical Science.
This journal is © The Royal Society of Chemistry 2016

Cubic Three–Dimensional Hybrid Silica Solids for Nuclear Hyperpo–larization

David Baudouin,[a] Henri A. van Kalkeren,[a] Aurélien Bornet, [b] Basile Vuichoud,[b] Laurent Veyre,[a] Matthieu Cavaillès,[a] Martin Schwarzwälder,[c] Wei-Chih Liao,[c] David Gajan,[d] Geoffrey Bodenhausen,[b,e,f,g] Lyndon Emsley,[b] Anne Lesage,[d] Sami Jannin,[b] Christophe Copéret,[c*] Chloé Thieuleux,[a*]

a Université de Lyon, Institut de Chimie de Lyon, LC2P2, UMR 5265 CNRS-CPE Lyon-UCBL, CPE Lyon, 43 Bvd du 11 Novembre 1918, 69100 Villeurbanne, France.

b Institut des Sciences et Ingénierie Chimiques, Ecole Polytechnique Fédérale de Lausanne (EPFL), Batochime, CH-1015 Lausanne, Switzerland.

c ETH Zürich, Department of Chemistry and Applied Biosciences, Vladimir-Prelog-Weg 1-5/10. 8093 Zürich, Switzerland..

d Centre de RMN à Très Hauts Champs, Institut de Sciences Analytiques (CNRS / ENS Lyon / UCB Lyon 1), Université de Lyon, 69100 Villeurbanne, France.

e Département de Chimie, Ecole Normale Supérieure, 24 Rue Lhomond, 75231 Paris Cedex 05, France.

f Université Pierre-et-Marie Curie, Paris, France.

g UMR 7203, CNRS/UPMC/ENS, Paris, France.

Preparation of 1/yy-N3-SBA-16 material. Representative procedure.

Procedure inspired from based from Arnaud Boullanger, Johan Alauzun, Ahmad Mehdi, Catherine Reyé, Robert J. P. Corriu, New J. Chem. 2010, 34, 738-743.

Preparation of 1/100_N_3_SBA-16 material. General procedure

Pluronic F127 (2g, ratio F127 to Si-based reactant: 4□10-3), NaCl (7.19, ratio F127 to Si-based reactant: 35 equiv) in aqueous 2 M HCl (20mL, 0.5 mL/mmol siloxy-precursors) and water (60mL, 1.5 mL/mmol siloxy-precursors) was dissolved by stirring it in a closed 250 mL teflon bottle (250 mL bottle used for 80 mL reaction volumes) at RT for 1.5 h and then for 1 h at 40 °C. Then, TEOS (8.31g, 100 equiv) and (3-azidopropyl)triethoxylsilane (99mg, 1 equiv) were simultaneously added. The bottle was closed again and vigorously stirred at 40 °C for 20 h before it was placed in a pre-heated oven at 100 °C for 2 h without agitation. The white precipitate was filtered and washed with EtOH (3 × 1/3 of initial reaction volume) and acetone (3 × 1/3 of initial reaction volume). The resulted solid was then crushed in a mortar and dried at 100 °C under vacuum (10-5 mbar) for 5 h. Then the material is stirred in water/pyridine solution (1:1, 1.5 mL/mmol siloxy-precursors) adjusted to pH 6.5 by aqueous HCl (2 M) at 70 °C for 16 h. Next, the material is filtered and washed with EtOH (3 × 1/3 of reaction volume) and acetone (3 × 1/3 of reaction volume) and dried at 100 °C under vacuum (10^{-5} mbar) for 15 h. 2.39g of material, yield: 96%.

Preparation of 1/140_N_3_SBA-16 material.

1/140-N_3-SBA-16 material was prepared following the above-described procedure using 8.34g of TEOS, 70.7mg of (3-azidopropyl)triethoxylsilane, 2g of F127, 7.19g of NaCl, 20mL of 2M HCl solution, 60mL of water, leading to 2.37g of solid.

Preparation of HYPSO-3 through Cu-AAC. Representative procedure.

Preparation of 1/100_HYPSO-3

Under an argon atmosphere, O-propargyl TEMPO (30, 342 mg, 2.67 mmol) was added to a suspension of 1/100_N_3_SBA-16 (20 g, 0.535 mmol azide) in DMF (20 mL) and Et3N (880 µL). Then a solution of CuI (3.1 mg, 16 µmol) in DMF/Et_3N (1:1, 240 µL) was added. The mixture was stirred for 72 h at 50 °C and then filtrated and washed with DMF (2 × 20 mL), EtOH (3 × 20 mL), Et_2O (2 × 20 mL). The product was then dried under vacuum (10^{-5} mbar) at 100 °C for 15 h.

CW EPR Spectroscopy (CW EPR)

Continuous Wave (CW) EPR spectra were recorded on a Bruker EMX X Band spectrometer (9.5 GHz microwave frequency). Conversion time was set to 40.96 ms, time constant to 5.12 ms respectively and 1024 data points were recorded. The modulation frequency was 100 kHz and the modulation amplitude 1 Gauss. In all measurements, attenuation was varied such that no saturation was observed.

Peak linewidth.

For each sample, the material was impregnated with 1,1,2,2-Tetrachloroethane and filled in a 3.0 mm quartz tube. All spectra were recorded at 110K using a nitrogen flow cryostat. Attenuation was varied from 32 to 20 dB. The EPR spectrum of a nitroxide radical consists of three lines due to strong hyperfine interaction with the 14N nucleus. For the line-width measurements, we have used the central line. Since it is least broadened by the g-tensor and hyperfine anisotropies, it is therefore the most sensitive to the dipolar broadening. For the obtained signal to noise ratios, the estimated linewidth errors were within the order of 5 %.

Spin count.

The samples were filled in a 3.0 mm quartz tube with a maximum sample height of 3mm. The sample position in the cavity was carefully optimized. The spectra were recorded at room temperature and with a sweep width of 600 Gauss and attenuation between 26 and 14 dB. The amount of radical was determined by double integration of the CW spectra and referencing to calibration curve of TEMPO in toluene solutions measured for the concentration range between 0.4 and 80 mM. An additional correction for the difference in the incident microwave power has been taken into account. Data was processed with OriginLab.

EPR analysis of filtered solutions after dissolution.

After dissolution, the solution was filtered and subjected to centrifugation (5000 rpm for 5 min). The supernatant was filled in a Hirschmann glass capillary with a sample height of 18 mm, which was then closed with putty. The sample position in the cavity was carefully optimized. The spectra were recorded at room temperature with a sweep width of 600 Gauss and attenuation at 20 dB. The number of scans per spectrum was adjusted to have a reasonable signal-to-noise ratio. The amount of radical was determined by double integration of the CW spectrum and referencing to the calibration curve of 4-hydroxy TEMPO aqueous solutions measured of the concentration range between 0.002 and 0.2 mM. An additional correction for the difference in the incident microwave power has been taken into account. Data was processed with MATLAB ® (R2011a, MathWorks Inc.).

Transmission Electron Microscopy (TEM).

Conventional TEM micrographs were performed at the "Centre Technologique des Microstructures", UCBL, Villeurbanne, France, using a JEOL 2100F electron microscope. The acceleration voltage was 200 kV. The samples were prepared by dispersing a drop of the ethanol suspension of a ground sample on a Cu grid covered by a carbon film.

Small Angle X-Ray Diffraction (SA-XRD).

Small-Angle X-ray scattering (SAXS) on powder was carried out with a Bruker D8 Avance diffractometer (33 kV & 45 mA) with CuKα radiation (λ = 0.154 nm) in the Service Diffraction RX, IRCE Lyon, France. The diffraction patterns were collected in the 2θ angle range [0.45°-7.0°] at a scanning rate of 0.1°/min. The interplane spacings, d(hkl) for different Miller indices (hkl) were calculated using the Bragg's law (nk = 2dsinθ). The lattice parameter (a0) for the hexagonal structured mesoporous material is given by a0 = 2d(100)/$\sqrt{3}$

Nitrogen Adsorption-desorption (BET).

The Nitrogen adsorption and desorption measurements were achieved at 77 K using a BELSORB-Max from BEL-JAPAN. Before N_2 adsorption, the samples were outgassed at 10^{-5} mbar at 408 K for 12 h. The pore diameter distribution and the mean pore diameter (d_p) were calculated using Barrett–Joyner–

Halenda (BJH) method. The specific surface area (S_{BET}) was according to Brunauer–Emmett–Teller (BET) equation.

MAS–DNP NMR Experiments.

Sample Preparation

Samples were prepared by incipient wetness impregnation (see the movie http://pubs.acs.org/JACSbeta/scivee/index.html#video2) using 8.5-16.2 mg of dry powder with 20-30 μL of an analyte containing solution or with pure solvent (H_2O or 1,1,2,2-tetrachloroethane, $EtCl_4$). The total mass of impregnated material was determined, and the sample was mixed using a glass-stirring rod to obtain a homogeneous distribution of the solution in the powder. The impregnated powder was then packed into a 3.2 mm sapphire NMR rotor to maximize MW penetration into the sample. The mass of impregnated material inside the rotor was determined, and a tight polyfluoroethylene plug was inserted to prevent any leakage of the solvent during spinning. The rotor was capped with a zirconia drive tip and quickly inserted into the DNP spectrometer.

DNP Solid-State MAS-NMR Spectroscopy.

All spectra were acquired on a Bruker Avance III 400 MHz DNP NMR spectrometer equipped with a 263 GHz gyrotron microwave system (B_0 = 9.4 T, $\omega^H/2\pi$ = 400 MHz, $\omega^C/2\pi$ = 100 MHz, $\omega^{Si}/2\pi$ = 79.5 MHz). The field sweep coil of the NMR magnet was set so that MW irradiation occurred at the DNP enhancement maximum of TOTAPOL (263.334 GHz), with an estimated 4 W power of the MW beam at the output of the probe waveguide. ^{1}H, ^{13}C, and ^{29}Si spectra were recorded using a triple resonance low-temperature CPMAS probe with a sample temperature of 99 K and sample spinning frequencies of 8 kHz for the ^{13}C and ^{29}Si spectra. SPINAL-64 heteronuclear decoupling was applied during acquisition ($\omega_1^H/2\pi$ = 100 kHz). ^{1}H, ^{13}C and ^{29}Si chemical shifts are referenced to TMS at 0 ppm. Standard cross-polarization (CP) was used for 1D carbon-13 and silicon-29 spectra. For ^{13}C CPMAS, the ^{1}H $\pi/2$ pulse length was 2.5 µs (100 kHz). A linear amplitude ramp (from 90% to 100% of the nominal RF field strength) was used for the ^{1}H channel, with a 2 ms contact time and a nominal RF field amplitude of ν_1 = 76 kHz for ^{1}H and 40 kHz for ^{13}C. SPINAL-64 proton decoupling was applied during the acquisition of the ^{13}C signal with an RF field amplitude of ν_1 = 100 kHz. The ^{13}C acquisition time was 20 ms with 1608 complex points.

For ^{29}Si CP-MAS, the ^{1}H $\pi/2$ pulse length was 2.5 µs (100 kHz). A linear amplitude ramp (from 90% to 100% of the nominal RF field strength) was used for the ^{1}H channel, with a 2 ms contact time and a nominal RF field amplitude of ν_1 = 76 kHz for ^{1}H and 35 kHz for ^{29}Si. SPINAL-64 proton decoupling was applied during the acquisition of the ^{29}Si signal with an RF field amplitude of ν_1 = 100 kHz. The ^{29}Si acquisition time was 20 ms with 1608 complex points.

Spectra were acquired with 32 - 256 scans for ^{13}C and 32-1024 scans for ^{29}Si in order to obtain a good signal on noise ratio.

Processing of the spectra was done using the Topspin software package.

Calculation of DNP Enhancement Factors (ε).

DNP enhancement factors on the nucleus X (ε_X) were determined by scaling the intensities of the spectra of the nuclei X obtained under the same experimental conditions with or without MW irradiation.

dDNP experiments

Sample preparation

A solution of 20 vol% H_2O and 80 vol% D_2O is prepared for ^1H polarization study. 20 to 25 mg of HYPSO is impregnated with this solution so that the volume of solution corresponds to 90-100 % of the HYPSO total pore volume ($P/P_0<0.99$), determined by N_2 adsorption at 77K. The impregnated material is then transferred to a home-built Teflon sample holder, then placed in the polarizer and cooled down to 4.2 K or 1.2 K.

Experimental method

4. DNP is performed at 4.2 K or 1.2 K by microwave irradiation (188.3 GHz, set for negative DNP, and 87.5 mW) in a field of 6.7 T (285.23 MHz for protons). Frequency modulation can be applied or not. When it was applied, the amplitude of the frequency modulation was set to $\Delta\nu_{\mu w}$ = 100 MHz with a modulation frequency f_{mod} = 10 kHz. The used setup is described in the experimental description.

5. The DNP build-up of ^1H spins is measured with 1° nutation angle pulses followed by an acquisition period of 1ms. The resulting free induction decay is Fourier transformed, phased and integrated to obtain a value of ^1H spin for the corresponding acquisition period. This sequence is applied every 5 seconds during at least 250 seconds (up to 1500 seconds for long build up time), thus, obtaining at least 50 values of ^1H spin.

Fitting parameters

6. The resulting DNP build-up curve is fitted with function defined in function of time (t) as

$$y(t) = P_{max}\left[1 - exp\left(-\frac{t}{\tau_{DNP}}\right)\right]$$

leading to the values P_{max}, and to the polarization build-up time τ_{DNP}. The fit is performed using the asymptotic-symmetry based method, chi-square minimization being the procedure used to minimize the residual sum of square (refer to "Inference in Nonlinear Fitting" guide line of origin lab for more details) between the experimental points and the calculated values. The ^1H polarization P_{max} hence obtained is used for comparing the materials and hereunder referred as $P(^1H)$. The build-up time τ_{DNP} (seconds) corresponds to time required to reach 63% of polarization, $5\times\tau_{DNP}$ corresponding to 99% of P_{max} = $P(^1H)$.

Table S1 – Organic function loadings and textural characteristics of the materials obtained by SAXS and N_2-adsorption desorption at 77K – HYPSO-3.

Materials	[≡SiR] $\mu mol_{\equiv SiR}.g^{-1}$	S_{BET} $m^2.g^{-1}$	V_p(tot.)[a] $m^2.g^{-1}$	V_p(μ.)[b] $m^2.g^{-1}$	D_{micro}[c] nm	D_{meso}[d] nm	X-Ray diffraction Structure	d(110)[e] nm	a_0[f] nm	$L_{\mu pore}$[g] nm
1/34_N_3_SBA-16	472	1012	0,68	0,31	1,7	6,2	cub.	11.2	15.8	9,6
1/34_HYPSO-3	472	729	0,50	0,19	1,6	5,4	cub.	11.2	15.8	10,4
1/60_N_3_SBA-16	272	1010	0,66	0,29	1,6	6,3	n.d.	n.d.	n.d.	n.d.
1/60_HYPSO-3	272	752	0,52	0,22	1,6	5,4	cub.	11.0	15.6	10,2
1/100_N_3_SBA-16	164	913	0,62	0,11	1,7	6,2	n.d.	n.d.	n.d.	n.d.
1/100_HYPSO-3	164	893	0,63	0,26	1,3	6,3	cub.	10.5	14,9	8,6
1/140_N_3_SBA-16	118	1184	0,82	0,33	1,4	7,0	n.d.	n.d.	n.d.	n.d.
1/140_HYPSO-3	118	983	0,69	0,26	1,7	7,1	cub.	11.6	16.4	9,3
1/320_N_3_SBA-16	52	1068	0,75	0,48	1,7	7,0	n.d.	n.d.	n.d.	n.d.
1/320_HYPSO-3	52	714	0,48	0,27	1,6	5,4	cub.	12,5	17,7	12,3

a- Total pore volume corresponding to the quantity of N_2 adsorbed at P/P_0=0,99.
b- Micropore volume, calculated from the α_S plot
c- Micropore mean diameter calculated using MP model;
d- Mesopore mean diameter calculated using BJH model (adsorption branch)
e- The interplane spacings of SBA-15 and SBA-16 are d(100) and d(110) respectively
f- The lattice parameter is given by $a0 = d(110)\times\sqrt{2}$.
g- Micropore mean length, calculated as: $=(d(110) *2/(\cos(\pi/4)-2* D_{meso})/2$

Table S2 – Organic function loadings and textural characteristics of the materials obtained by SAXS and N_2-adsorption desorption at 77K – HYPSO-2.

Materials	[≡SiR] $\mu mol_{\equiv SiR}.g^{-1}$	S_{BET} $m^2.g^{-1}$	V_p(tot.)[a] $m^2.g^{-1}$	V_p(µ.)[b] $m^2.g^{-1}$	D_{micro}[c] nm	D_{meso}[d] nm	X-Ray diffraction Structure	d(100)[e] nm	a_0[f] nm	t_{wall}[g] nm
1/34_HYPSO-2	472	832	1,15	0,05	1,8	8,1	hexagonal	11	12,7	4,6
1/60_HYPSO-2	272	767	1,07	0,04	1,9	8	hexagonal	10,8	12,4	4,4
1/100_HYPSO-2	164	866	1,19	0,09	1,9	9,2	hexagonal	10,8	12,4	3,2
1/140_HYPSO-2	118	849	1,15	0,05	1,9	8	hexagonal	10,5	12,1	4,1

a- Total pore volume corresponding to the quantity of N_2 adsorbed at P/P_0=0,99.
b- Micropore volume, calculated from the α_S plot
c- Micropore mean diameter calculated using MP model;
d- Mesopore mean diameter calculated using BJH model (adsorption branch)
e- The interplane spacings of SBA-15 and SBA-16 are d(100) and d(110) respectively
f- The lattice parameter is given by $a0 = d(110)\times\sqrt{2}$.
g- Micropore mean length, calculated as: $=(d(110) *2/(\cos(\pi/4)-2* D_{meso})/2$

As shown in Table 2, CuAAC yields vary from 88 to 64 % and decreases slowly with decreasing 1/xx ratio. Note that the same reaction on 1/100 and 1/140_N_3_SBA -15 gave CuAAC yield of 87-88 %, indicating a slightly greater accessibility of the low concentrated azido in SBA-15 pores, possibly because of the larger pore structure of SBA-15 vs. SBA-16 type materials.

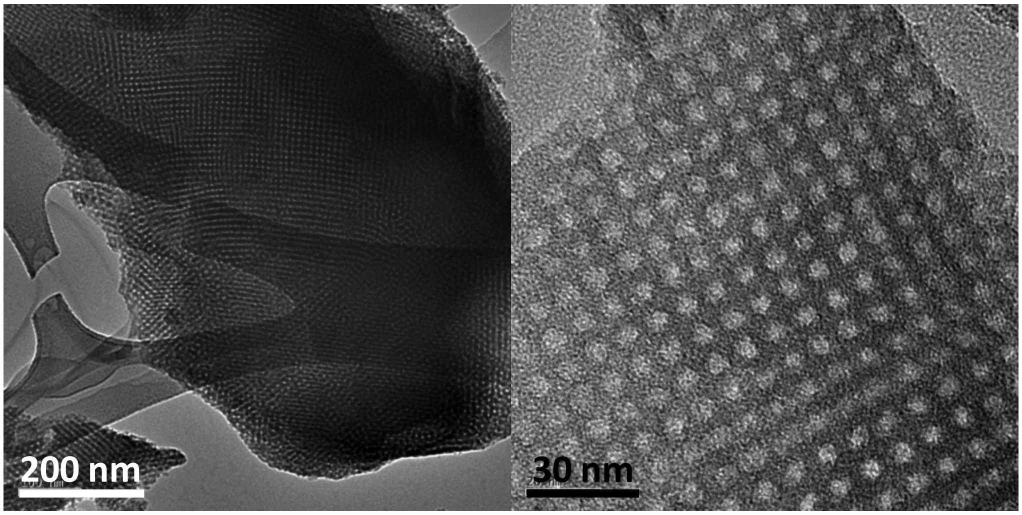

Figure S1. TEM pictures of 1/34_N_3_SBA-16 in the {100} plane at different magnifications.

Figure S2 – Schematic representation of HYPSO-3 preparation and physical features.

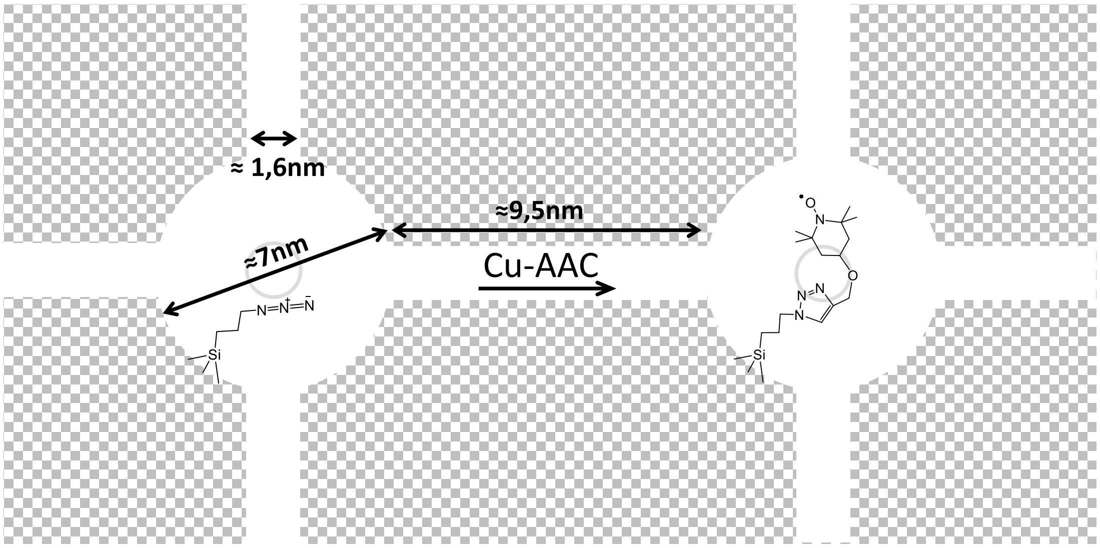

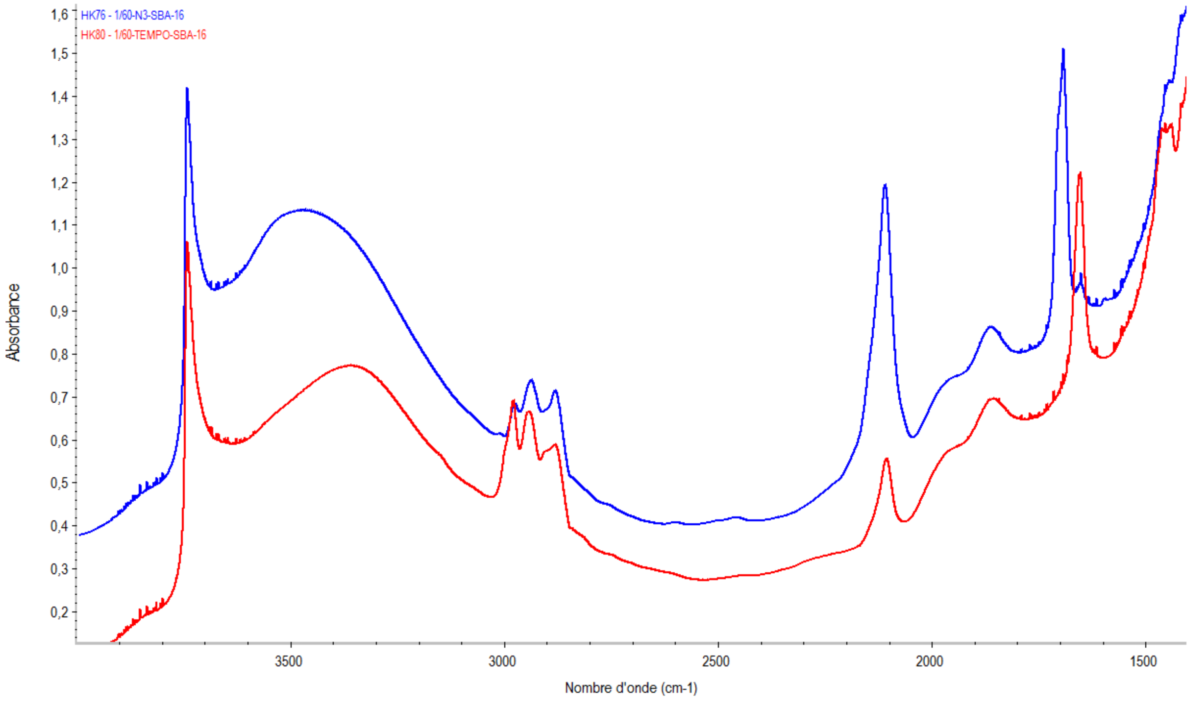

Figure S3. DRIFT spectra of 1/60_N_3_SBA-16 and 1/60_HYPSO-3.

The peak at ca. 2100 cm^{-1} corresponds to $–N_3$ stretching vibrations.

Diffusive Reflectance Infra-Red Fourier Transformed (DRIFT) analysis of the powder allowed to evaluate the efficiency the cycloaddition on HYPSO-3 (referred to as CuAAC yield) by monitoring the decrease of the intensity of the $-N_3$ absorption peak, at 2210 cm^{-1} (see Figure S3).

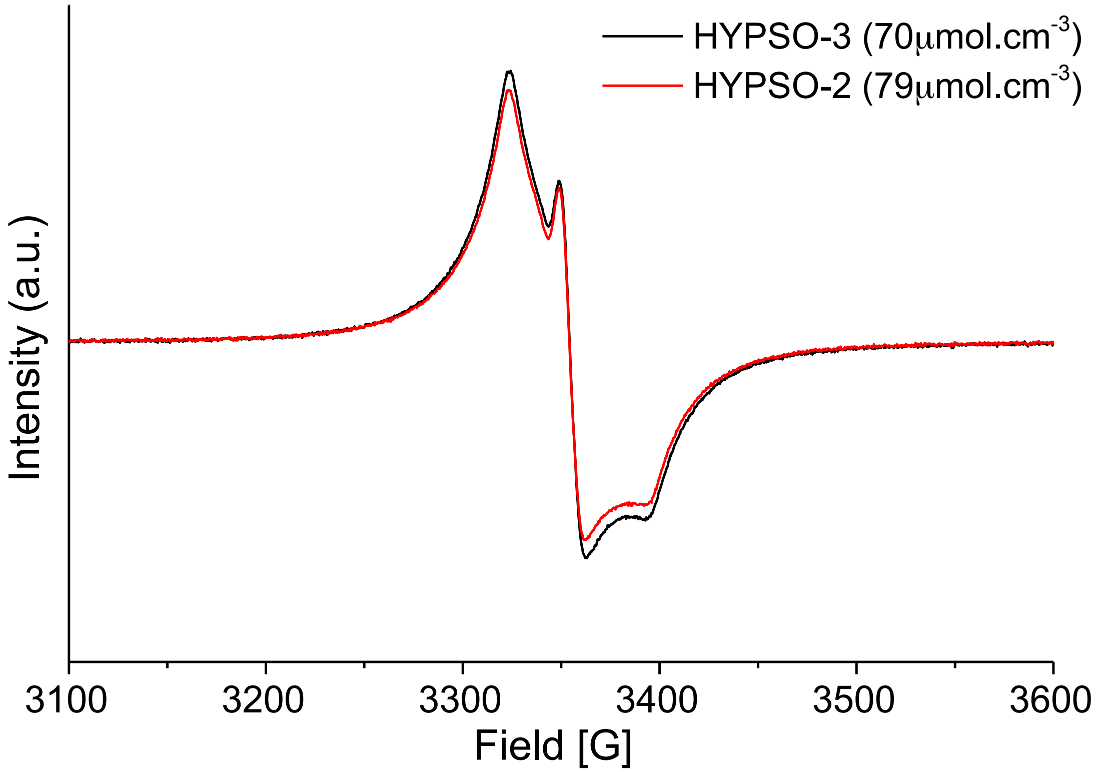

Figure S4 – EPR spectra at room temperature of HYPSO-2 and -3 having a radical concentration of 79 and 70$\mu mol_{NO}.cm^{-3}$.

Table S3 – Chemical and radical characteristics of HYPSO-2 and -3 materials.

Materials	[≡SiR] *$\mu mol_{\equiv SiR}.g^{-1}$*	[NO•] *$\mu mol_{NO}.g^{-1}$*	Cu_{AAC} yield[a] *%*	EPR yield[b] *%*	[NO•][c] *$\mu mol_{NO}.cm^{-3}$*	Line width *G*
1/34_HYPSO-3	472	320	88	68	640	20,9
1/60_HYPSO-3	272	140	81	52	269	15,3
1/100_HYPSO-3	164	78	77	47	124	13,1
1/140_HYPSO-3	118	44	74	37	64	12,1
1/320_HYPSO-3	52	33	64	63	67	n.d.
1/34_HYPSO-2	472	234	n.d.	49	203	16,8
1/60_HYPSO-2	272	115	n.d.	52	108	13,5
1/100_HYPSO-2	164	94	88	52	79	12,7
1/140_HYPSO-2	118	53	87	35	46	12,1

a- Percentage of $-N_3$ reacted after Cu-AAC
b- Percentage of $NO^•$ present with regard to the initial $-N_3$ present
c- The concentration is given per unit of volume calculated using the total pore volume (P/P_0=0,99)

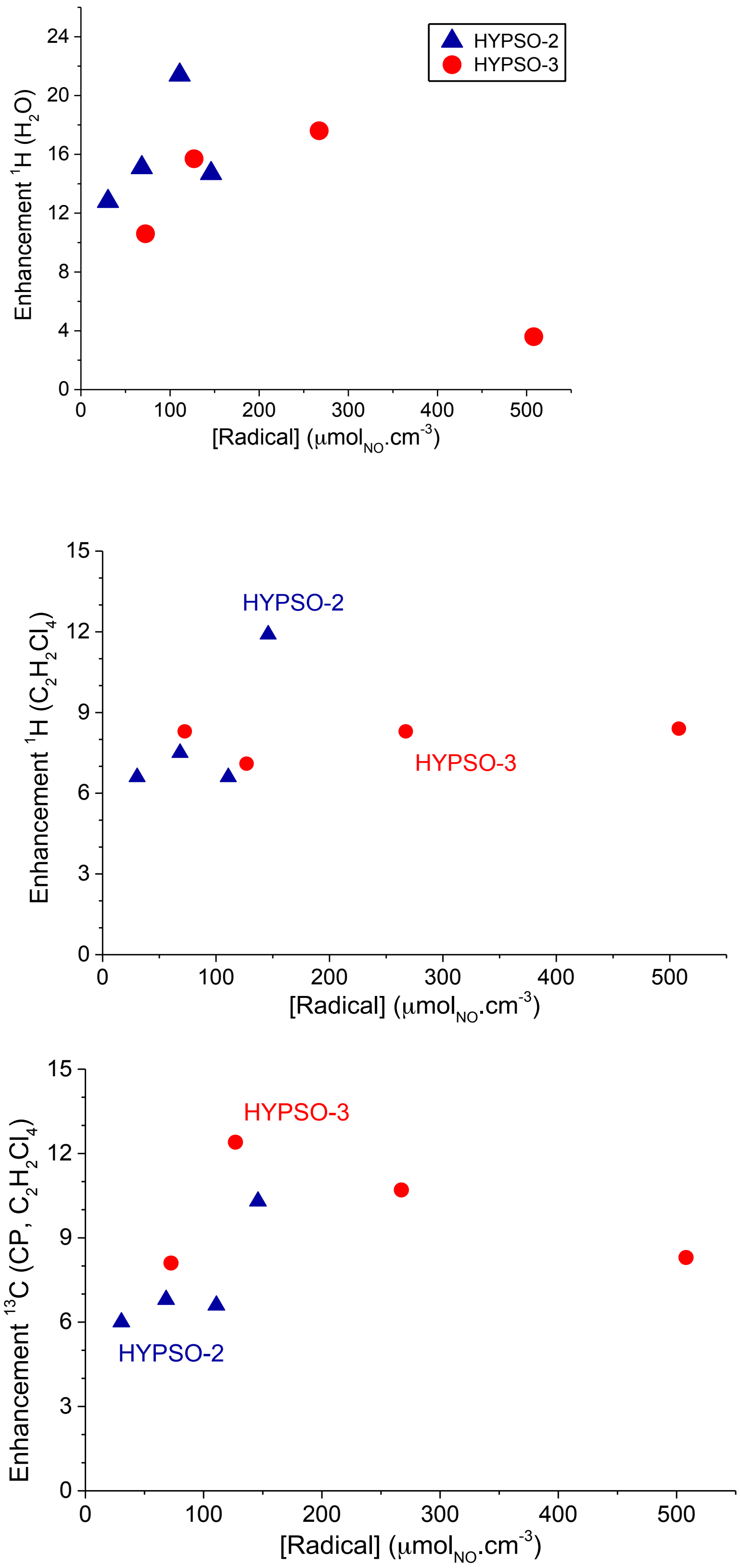

Figure S5. Enhancements factors of H_2O and $C_2H_2Cl_4$ impregnated in HYPSO-2 & -3, measured at 99K in DNP-MAS measurements. ^{13}C enhancement was measured under cross polarization conditions.

Worthy of note the proton enhancement ε_H observed for HYPSO-3 impregnated with $C_2H_2Cl_6$ remains stable at ca. 8.5 for all the radical concentrations studied, while HYPSO-2 enhances the signal better when the radical concentration is above 100 $\mu mol_{NO}.cm^{-3}$. The enhancement ε_C obtained by

CP on the ^{13}C of the solvent presents a volcano-shaped curve with a maximum at ca. 130 $\mu mol_{NO}.cm^{-3}$ (ε_C=12.5), with values slightly greater for HYPSO-3 than for HYPSO-2. Overall, HYPSO-3 leads to ^1H and ^{13}C enhancements using H_2O and $C_2H_2Cl_4$ comparable to HYPSO-2. In addition in water the range of optimum radical concentration for HYPSO-3 is significantly greater than HYPSO-2.

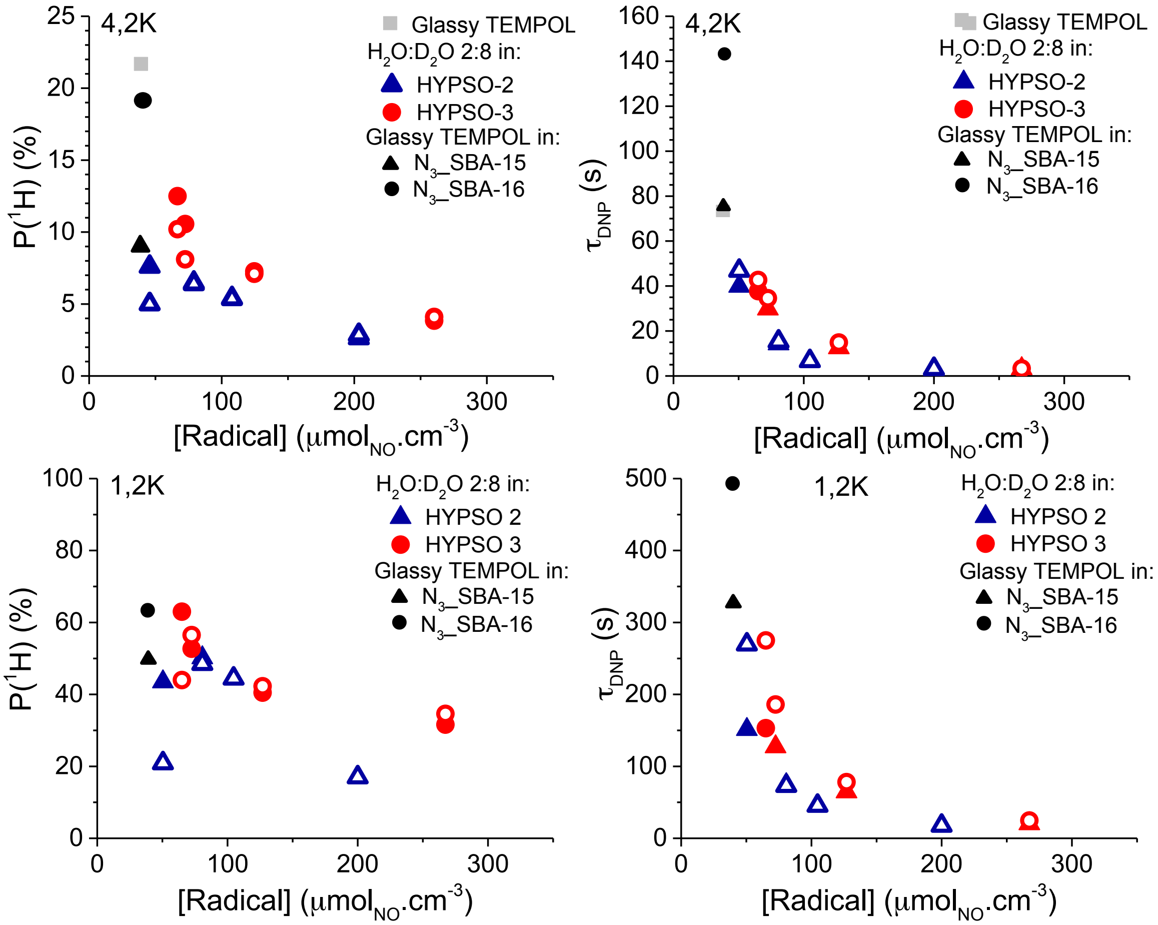

Figure S6. Left: Evolution of P(1H) with the HYPSO-2 (red triangle) and HYPSO-3(blue circles) radical concentration (in μmolNO.cm-3) at 4.2 and 1.2K. The data obtained with frequency modulation are full symbols, the empty symbols were obtained without modulation. Right: corresponding build up time constant using mono-exponential fitting. All data reported were recorded with modulation. At 4.2K: the grey squares correspond to TEMPOL in "glassy" H2O:D2O:Glycerol-d8 (10:40:50) mixture. The open circles and triangles correspond to an optimal 40mM TEMPOL "glassy" solution of H_2O:D_2O:Glycerol-d8 (10:40:50) mixture impregnated in 1/140_N3_SBA-16 and 1/140_N3_SBA-15, respectively.

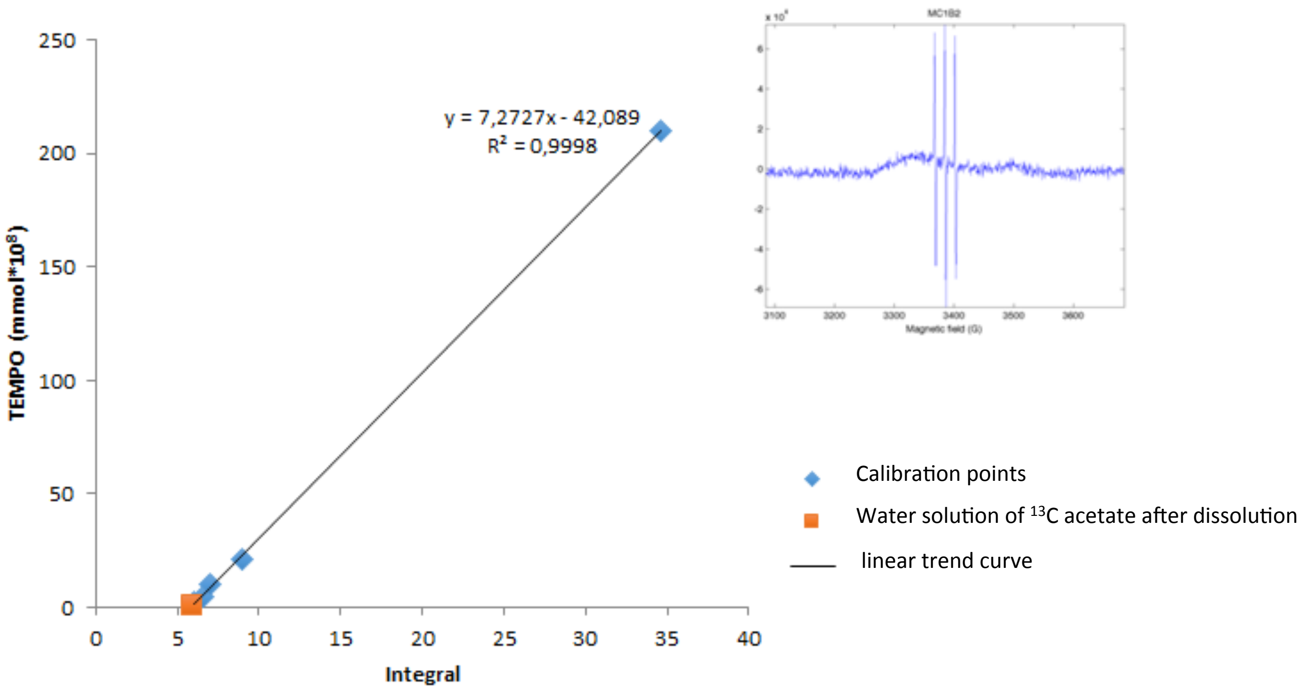

Figure S7. Left: ESR titration of residual TEMPO contained in the sodium [1-^{13}C]-acetate water solution after dissolution (orange square) using a calibration curve (calibration points as blue diamonds); Right: ESR signal of residual TEMPO contained in the sodium [1-^{13}C]-acetate solution after dissolution.
